# All That Glitters is not Gold: Apical Hypertrophic Cardiomyopathy Mimicking Acute Coronary Syndrome

**DOI:** 10.4021/cr180w

**Published:** 2012-05-20

**Authors:** Umashankar Lakshmanadoss, Abhishek Kulkarni, Shobana Balakrishnan, Nidhi Shree, Kishore Harjai, Dinesh Jagasia

**Affiliations:** a Division of Cardiology, Department of Medicine, Guthrie Health System, Sayre, PA, 18840, USA; b Division of Hospital Medicine, Johns Hopkins Bayview Medical Center, Baltimore, MD, USA

**Keywords:** Hypertrophic cardiomyopathy, Acute coronary syndrome, Apical hypertrophic cardiomyopathy

## Abstract

Hypertrophic cardiomyopathy is characterized by the idiopathic hypertrophy of the left ventricle (and occasionally right ventricle). HCM is an autosomal dominant disease, with variable penetration. In Asian population, apical hypertrophic cardiomyopathy is relatively common (25%). However, this is relatively rare in Caucasian population (0.2%). Patients with HCM, often presents with typical exertional chest pain and shortness of breath. Apical HCM patients tend to have milder symptoms. However, the clinical presentation and electrocardiographic features of Apical HCM often mimic acute coronary syndrome and high index of suspicion is warranted in differentiating this condition. Patients with apical HCM have relatively better prognosis when compare to the other varieties. Here, we are presenting a patient who presented with typical exertional chest pain whose electrocardiographic changes are concerning for acute ischemic changes.

## Case Report

**Figure 1:**
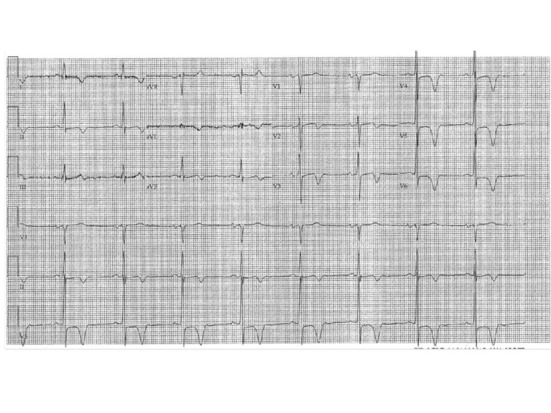
Diffuse deep T wave inversion in anterolateral leads (L1, aVL, V2 - V6).

**Figure 2:**
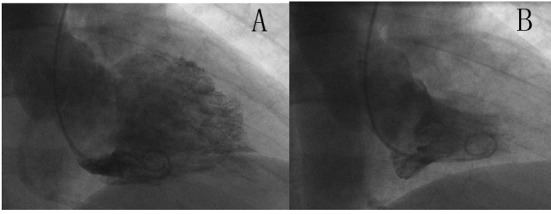
A: Left ventriculogram in Diastole; B: Left ventriculogram in Systole - (“ace-of-spades” sign).

**Figure 3:**
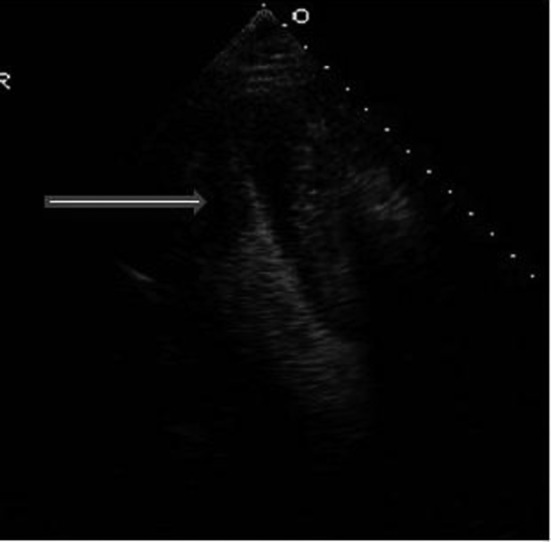
Transthoracic echocardiogram showing the apical hypertrophy of the left ventricle.

A 68-year-old male patient presented to the emergency department due to history of exertional chest pain for the past few weeks. His chest pain was central in location, described as a pressure like sensation with severity of 4/10 in intensity, radiating to the left shoulder, brought up with exertion and relieved with rest within 5 minutes. He also noted to have paroxysms of palpitations for the past one month, brought up by exertion. He denied any history of orthopnea, paroxysmal nocturnal dyspnea and lightheadedness. His past medical history is significant for essential hypertension, paroxysmal atrial fibrillation, transient ischemic attack, bleeding duodenal ulcer s/p cauterization, s/p argon photo coagulation of arterio venous malformations of the ascending colon and benign prostate hypertrophy. His medications include Diltiazem 90 mg, Lisinopril 40 mg, Metoprolol XL 50 mg and Pantoprazole 40 mg daily. His CHADS_2_ score was 3. However, due to his history of gastrointestinal bleeding, he is not on any anticoagulation or antiplatelet therapy. His pulse rate is 62/min; regular in rhythm; no jugular venous distension; auscultation is notable for S4 gallop; no murmurs heard. His resting electrocardiogram revealed deep symmetrical T wave inversion in anterolateral leads and voltage criteria for left ventricular hypertrophy (Fig. 1). His complete blood count and basic metabolic profile are normal. His cardiac enzymes are negative consecutively. Due to his classical history and electrocardiographic changes, he had cardiac catheterization. His coronaries are with no obstructive disease. His left ventriculography revealed complete systolic obliteration towards the leftventricular apex and a small ventricular cavity at the base (“ace-of-spades” sign) (Fig. 2A, 2B). His transthoracic echocardiogram was consistent with apical hypertrophy of the left ventricle (Fig. 3). He was diagnosed to have apical hypertrophic cardiomyopathy. His tele monitor revealed multiple supraventricular tachycardia and nonsustained ventricular tachycardia. He was continued on his home medications including Diltiazem and Metoprolol.

## Discussion

Hypertrophic cardiomyopathy (HCM) is a clinical syndrome characterized by an increased myocardial mass with a resultant small ventricular cavity. It is the most common hereditary cardiac disease and the most frequently found cardiomyopathy [1]. It is a “disease of the sarcomere” which leads to the disarray of the myocardial fibers. Apical hypertrophic cardiomyopathy is a form of non-obstructive hypertrophic cardiomyopathy localized to the left ventricular apex that was first described in Japanese patients with precordial deep T wave inversions (referred to as giant T wave inversions) in 1976 [2]. This condition is common in Japan and estimated to represent 25% of Japanese patients with HCM [3-5]. It is otherwise called as Yamaguchi's disease. However, it has been identified in 1% to 2% of patients with hypertrophic cardiomyopathy in Caucasians [6]. Patients can present with symptoms of angina and dyspnea with a predominant exertional component. Myocardial ischemia could happen in the presence of normal coronaries in angiogram. Although the exact mechanism is unknown, the underlying pathophysiology is a mismatch of supply and demand secondary to small - vessel coronary artery disease with decreased vasodilator capacity, delayed relaxation of myocardium, decreased capillary-to-myocardial fiber ratio and decreased coronary perfusion pressure [8]. Palpitations and syncope should prompt further evaluation of arrhythmias. Atrial arrhythmias (from atrial dilation) and serious ventricular arrhythmias have been described in patients with apical HCM. Overall, patients with apical HCM tend to have milder symptoms.

Electrocardiograms reveal left ventricular hypertrophy and giant T wave inversion defined as T inversion > 10 mm. In patients with apical hypertrophic cardiomyopathy, a transthoracic echocardiogram may not show the hypertrophy localized to the apex. Diastolic dysfunction is common due to ventricular hypertrophy. Transesophageal echocardiogram could be useful in demonstrating the apical thickening. In some cases apical hypertrophy may be confused with apical thrombus. This could be differentiated using the myocardial contrast echocardiogram using Perflutren Lipid Microsphere (Definity^®^). If echocardiographic images are inadequate, cardiac magnetic resonance imaging may be used to diagnose apical hypertrophic cardiomyopathy. Coronary angiogram may reveal underlying co existent coronary artery disease. Left ventriculography reveals the characteristic spade-like configuration of the left ventricle in end-diastole (due to the concentric hypertrophy of the apical myocardium), with obliteration of the apical cavity in end-systole (due to the vigor with which the hypertrophied myocardium contracts) [6]. During ventriculography, assessment of outflow tract obstruction should be done at rest or with provocation (isoproterenol, extra systoles, amyl nitrite). Patients with AHCM who have myocardial infarctions often lack angiographic evidence of significant coronary artery disease, with infarction perhaps caused by impaired coronary flow reserve, decreased capillary myocardial ratio, or small vessel disease [7].

The usual course of AHCM will be dictated by three adverse pathways: sudden cardiac arrest, congestive heart failure with preserved ejection fraction and atrial arrhythmias and it's complications including stroke. Unlike patients with hypertrophic obstructive cardiomyopathy, patients with AHCM generally have benign outcomes, and sudden cardiac death is rare. North American patients with AHCM have low long term cardiovascular mortality, but nearly one third of patients have increased cardiovascular morbidity. Presence of “giant” T wave inversion in the Japanese HCM patients has been identified as a predictor of favorable outcome [8]. However, the same couldn't be studied in North American population. Symptoms of angina and exertional dyspnea are well controlled with medical therapy with agents like beta blockers and calcium channel blockers.

### Conclusions

The correct diagnosis of apical HCM is of major importance because many of these patients present with typical chest pain and dramatic T wave inversions that frequently result in hospitalization for suspected coronary artery disease. Apical HCM has a benign prognosis in terms of cardiovascular mortality. One third of population could develop adverse clinical events including myocardial infarction, congestive heart failure and atrial arrhythmias.
